# Surface-Enhanced Raman Scattering Activity of ZrO_2_ Nanoparticles: Effect of Tetragonal and Monoclinic Phases

**DOI:** 10.3390/nano11092162

**Published:** 2021-08-24

**Authors:** Mingyue Yi, Yu Zhang, Jiawen Xu, Dingyuan Deng, Zhu Mao, Xiangchun Meng, Xiumin Shi, Bing Zhao

**Affiliations:** 1 College of Chemical Engineering, Changchun University of Technology, Changchun 130012, China; yimingyue95@163.com (M.Y.); zhangyjy0@163.com (Y.Z.); xujiawen20210719@163.com (J.X.); dengdingyuan0824@163.com (D.D.); mengxiangchun@ccut.edu.cn (X.M.); 2 School of Chemistry and Life Science, Changchun University of Technology, Changchun 130012, China; maozhu@ccut.edu.cn; 3 State Kay Laboratory of Supramolecular Structure and Material, Jilin University, Changchun 130012, China

**Keywords:** SERS, ZrO_2_, M phase, T phase, charge transfer

## Abstract

The effect of the ZrO_2_ crystal form on surface-enhanced Raman scattering (SERS) activity was studied. The ratio of the tetragonal (T) and monoclinic (M) phases of ZrO_2_ nanoparticles (ZrO_2_ NPs) was controlled by regulating the ratio of two types of additives in the hydrothermal synthesis method. The SERS intensity of 4-mercaptobenzoic acid (4–MBA) was gradually enhanced by changing the M and T phase ratio in ZrO_2_ NPs. The degree of charge transfer (CT) in the enhanced 4–MBA molecule was greater than 0.5, indicating that CT was the main contributor to SERS. The intensity of SERS was strongest when the ratio of the T crystal phase in ZrO_2_ was 99.7%, and the enhancement factor reached 2.21 × 10^4^. More importantly, the proposed study indicated that the T and M phases of the ZrO_2_ NPs affected the SERS enhancement. This study provides a new approach for developing high-quality SERS substrates and improving the transmission efficiency of molecular sensors.

## 1. Introduction

After the phenomenon of surface-enhanced Raman scattering (SERS) was first reported in the mid-1970s, it attracted the attention of many scholars. Researchers have published many articles related to SERS [1,2]. As a fairly new spectral detection technology, SERS has been widely used in the fields of chemistry, physics, biology, and medicine because of its high sensitivity, nondestructive detection, molecular fingerprint information, rapid and simple operation, etc. [3,4,5]. Single molecule SERS was studied by the Kneipp [6] and Nie [7] research groups in 1997, in which the enhancement factor (EF) reached up to 10^14^. Nowadays, electromagnetic mechanism (EM) and chemical mechanism (CM) are widely used to explain the enhancement mechanisms of SERS [8,9,10]. Recently, with the evolution of SERS research, SERS substrates have changed from the original precious metals (Au, Ag and Cu) [11,12,13] and the expensive metals (Pt and Pd) [14,15] to semiconductor materials (ZnO, CuO, TiO_2_) [16,17,18]. Semiconductor materials have good optics, electrics, biocompatibility, high stability, and low cost compared to the metal substrates. Therefore, they have greater research potential and application prospects [19].

A key problem in SERS research is the selection of suitable substrates and the optimization of the preparation process. Many studies have reported that zirconium dioxide (ZrO_2_) has excellent properties, such as high temperature, corrosion, and oxidation resistance, thermal and chemical stability, which ensures it is widely used in the production of artificial teeth, high temperature materials, electronics and bio ceramics [20,21,22]. Specifically, nanosized ZrO_2_ has three types of crystal structure: monoclinic (M), tetragonal (T) and cubic (C) [23,24], in addition to being an N-type semiconductor. Moreover, nano-ZrO_2_ exhibits surface and interface, quantum size, and macroscopic quantum tunneling effects [25]. Due to the small particle size, large specific surface area and incomplete coordination of atoms on the surface, there is an increase in the quantity of active sites on the nano-ZrO_2_ surface [26]. The electrons and holes, which are produced by nano-ZrO_2_ crystal particles under illumination, have strong reduction and oxidation ability as well as high photoelectric conversion efficiency [27]. The application scope of semiconductor materials is expanding with the development of the semiconductor technique. The use of semiconductor materials as SERS substrates is prevalent; however, to date, only two studies describing the use of ZrO_2_ as a SERS substrate have been reported. The crystalline form of nano-ZrO_2_ is mainly the M phase [24,28] and the EF can reach 10^3^. Nano-ZrO_2_ is very suitable as a SERS substrate due to its superior characteristics, and using nano-ZrO_2_ expands the range of semiconductor nanomaterials as SERS substrates. Meanwhile, it also leads to new applications of nano-ZrO_2_, which lay a foundation for the further research of the high-quality material in the application of nano sensor devices. More suitable preparation techniques of nano-ZrO_2_ are developed by improving the preparation process. The internal relationship between the SERS properties of a substrate and its structure is a contentious issue in SERS research.

In this work, nano-ZrO_2_ was prepared by hydrothermal synthesis and the SERS of 4-mercaptobenzoic acid (4–MBA) adsorbed on ZrO_2_ nanoparticles (NPs) was determined. The different crystal forms of ZrO_2_ NPs can be prepared by adding different types of additives in the hydrothermal synthesis. More interestingly, the proportion of the different crystal forms of ZrO_2_ NPs can be controlled by regulating the proportion of two types of additives. We found that there is an interdependence between the crystal form of ZrO_2_ NPs and the intensity of the SERS signal.

## 2. Materials and Methods

### 2.1. Chemicals

Zirconium chloride octahydrate (ZrOCl_2_·8H_2_O), 1,2-dichloroethane, and diethanolamine were purchased from Shanghai McLean Biochemistry Co., LTD. (Shanghai, China). Ammonia water (25%) and 4–MBA were purchased from Tianjin Furui Fine Chemical Co., LTD (Tianjin, China) and Sigma-Aldrich International Limited (Shanghai, China). Deionized water was used throughout the experiments. All reagents used were analytical grade and were used as supplied for sample preparation.

### 2.2. Preparation of ZrO_2_ NPs

ZrO_2_ NPs were prepared using the hydrothermal synthesis method according to the literature [29]. ZrOCl_2_·8H_2_O (3.9710 g) was added to 12 mL of deionized water and dissolved completely with stirring. Then, ammonia solution (10%) was added to the solution until the pH was approximately 7, and a white precipitate was produced. The precipitate was filtered repeatedly and washed with deionized water. The precipitate was transformed into a homogeneous suspension in deionized water, and the pH was adjusted to 9 with the addition of ammonia solution (5 wt %). The concentration of zirconium ions was then adjusted to 0.6 mol/L. Diethanolamine and 1,2-dichloroethane were utilized as a mixed additive to form the different crystal structures of ZrO_2_ NPs. The mixing ratios were 1:0, 4:1, 3:1, 2:1, 1:1, 1:2, 1:3, 1:4 and 0:1, respectively. One of the nine ratios of mixed additives was added to each sample of the suspension. The volume ratio of the additives to the suspension was 1:30. The volume of the suspension was adjusted to 16 mL using deionized water. The reaction solution was then stirred for 40 min and placed in a high-pressure hydrothermal reaction vessel. Nine groups of reactors were heated at 180 °C for 72 h. After the reaction, the synthesized ZrO_2_ was filtered, washed several times, and dried at room temperature.

### 2.3. Adsorption of Probe Molecules for SERS Measurement

ZrO_2_ (30 mg) with different T phase ratios was dispersed in 15 mL 4–MBA (1 × 10^−3^ mol/L) ethanol solution. The configured ZrO_2_ suspension was magnetically stirred for 8 h at room temperature. After centrifugation, ethanol and deionized water were used to wash the residue trice, and the surface modification of ZrO_2_ NPs was obtained.

### 2.4. Instrumentation

The crystal structure of all the samples was determined using Rikagu Smartlab X-ray diffraction patterns (Tokyo, Japan), with a scanning angle range of 10°–75°, and a scanning speed of 3°/min. SERS spectra were recorded by a Horiba HR Evolution Raman spectrometer (Paris, France) under 532 nm (or 2.33 eV) excitation. All Raman spectra were calibrated using Si plates. The penetration depth and diameter of the laser spot was approximately 10 and 1.3 µm, respectively. Scanning electron microscopy (SEM) was employed to study the size distribution of ZrO_2_ NPs using a JEOL-7610P (Tokyo, Japan). Ultraviolet-visible (UV-Vis) diffuse reflectance spectra (DRS) of the ZrO_2_ samples were recorded on a Cary 5000 UV-Vis spectrophotometer (Santa Clara, CA, USA) equipped with an integrating sphere using BaSO_4_ as a reference.

## 3. Results and Discussion

### 3.1. Characterization of ZrO_2_ NPs

**Figure 1:**
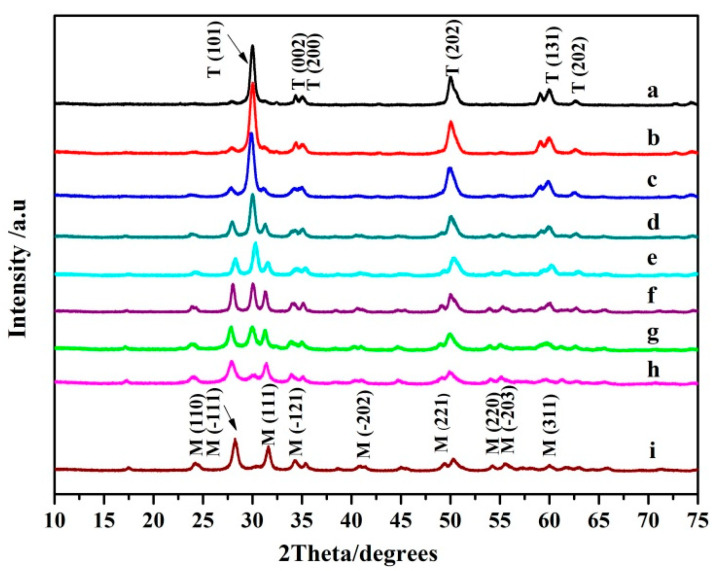
XRD patterns of ZrO_2_ NPs prepared with different ratios of diethanolamine and 1,2-dichloroethane as mixed additives. (**a**–**i**) correspond to the mixing ratio of 1:0, 4:1, 3:1, 2:1, 1:1, 1:2, 1:3, 1:4, and 0:1, respectively.

Previous reports [29,30] describe the preparation of ZrO_2_ NPs with different crystal forms using different additives during hydrothermal synthesis. Diethanolamine with its strong chelating ability encourages the formation of T–ZrO_2_ crystals, whereas alkyl halide 1,2-dichloroethane encourages the formation of M–ZrO_2_ crystals. Figure 1 shows the XRD diagram of ZrO_2_ NPs prepared by a hydrothermal method using diethanolamine and 1,2-dichloroethane as a mixed additive, and the mixing ratios are 1:0; 4:1; 3:1; 2:1; 1:1; 1:2, 1:3, 1:4, and 0:1, respectively. As shown in Figure 1, there are obvious differences in the crystal form of nano-ZrO_2_ prepared with pure diethanolamine or 1,2-dichloroethane as dispersant. The XRD diffraction curve when using diethanolamine as an additive is shown in Figure 1a, the characteristic Bragg reflections for the T phase: T (101), T (200), T (202) and T (131) (PDF card: 50–1089) are respectively at approximately 2θ = 30.45°, 35.30°, 50.26° and 60.2°. This indicates that the phase of ZrO_2_ is basically pure T phase [31]. However, the XRD diffraction curve when using 1,2-dichloroethane as an additive is shown in Figure 1i, the characteristic Bragg reflections for the M phase: M (111) and M (−111) (PDF card: 37-1484) are at approximately 2θ = 28.37° and 31.50°, respectively. This indicates that the ZrO_2_ crystal phase is basically pure M. Figure 1b–h are the XRD of ZrO_2_ NPs prepared using the diethanolamine and 1,2-dichloroethane mixture, in the mixing ratios stipulated above. We concluded that the intensity of the Bragg reflection of the T phase characteristic in the diffraction line (Figure 1b–h) decreases gradually, whereas the Bragg reflection of the M phase characteristic increases gradually with the decrease diethanolamine and the increase 1,2-dichloroethane. The particle size of ZrO_2_ NPs was calculated using the Scherrer formula: D = kλ/(β cos θ) [32] and is approximately 15.5–16.7 nm. The particle size of the ZrO_2_ NPs was confirmed using the SEM image (Figure S1 and Table S1) and was consistent with the results from the XRD.

It is necessary to calculate the T phase content in the sample ZrO_2_. To determine the correlation between the mixing ratio of diethanolamine and 1,2-dichloroethane additives, and the crystal form of the synthesized ZrO_2_ NPs. The T phase and M phase of ZrO_2_ can be identified by the most characteristic Bragg reflection of the XRD diffraction lines of all the samples: M (−111), T (101) and M (111). There were two M peaks (−111) and (111), and one T peak (101) in the sample. The T phase content in ZrO_2_ was calculated by the relative intensities. First, the strength of the integral of the three Bragg reflections: M (−111), T (101), and M (111) of the samples were extracted. Then we calculated the ratio of the T phase using the empirical expressions of Garvie and Nicholson [33,34]. The Formulas (1) and (2) are as follows:

(1) $$X_{t}=\frac{I_{t}(101)}{\left[I_{t}(101)+I_{m}(-111)+I_{m}(111)\right]}$$

(2) $$\nu_{t}=\frac{1.311X_{t}}{\left(1+0.311X_{t}\right)}$$

where ν_t_ is the volume fraction of the T phase, X_t_ is the integrated intensity ratio; I_m_ (111) and I_m_ (−111) are the (111) and (−111) intensities of the M phase of ZrO_2_, and I_t_ (101) is the (101) intensity of the T phase of ZrO_2_, respectively.

The T phase content of the ZrO_2_ crystal in Figure 1a–i were calculated by Formulas (1) and (2). The ratio of the T phase in the ZrO_2_ nanoparticles are 99.7%, 81.3%, 70.6%, 63.5%, 41.1%, 29.8%, 19.4%, 11.3%, and 2.5% corresponding to Figure 1a–i, respectively. The results show that ZrO_2_ with different crystal forms can be synthesized by different types of organic additives in the process of hydrothermal synthesis. The T phase content of ZrO_2_ NPs can be easily controlled by adjusting the ratio of diethanolamine to 1,2-dichloroethane. The content of the T phase of ZrO_2_ NPs gradually decreased, with decreasing diethanolamine and increasing 1,2-dichloroethane (Figure 1).

### 3.2. Raman Spectra of ZrO_2_ NPs

**Figure 2:**
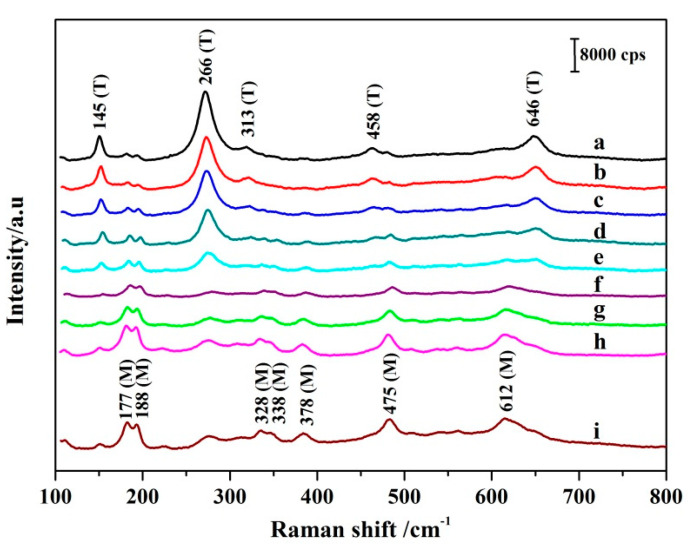
Raman spectra of ZrO_2_ NPs prepared with different ratios of diethanolamine and 1,2-dichloroethane as mixed additives. (**a**–**i**) correspond to the mixing ratio: 1:0, 4:1, 3:1, 2:1, 1:1, 1:2, 1:3, 1:4, and 0:1, respectively.

The Raman spectra of the synthesized ZrO_2_ NPs are shown in Figure 2. In general, Raman spectra of molecules appear due to the vibrational characteristics of different crystal phases of ZrO_2_. The Raman shifts at less than 800 cm^−1^ were assigned to the ZrO_2_ NPs. There were a few distinct vibrational bands located at 145, 266, 313, 458 and 646 cm^−1^ which correspond to the vibrational modes of the T phase of ZrO_2_ (Figure 2a). For the T phase of ZrO_2_, 6 modes (1A_1g_ + 3E_g_ + 2B_1g_) are Raman active [35]; the E representations are two dimensional whereas the other modes are one dimensional. This suggests that the ZrO_2_ is essentially pure T phase when diethanolamine is used as an additive. However, there were a few distinct vibrational bands located at 177, 188, 328, 338, 378, 475, and 612 cm^−1^ which correspond to the vibrational modes of the M phase of ZrO_2_ (Figure 2i). For the M phase of ZrO_2_, 18 modes (9A_g_ + 9B_g_) are Raman active [36]. This indicates that the ZrO_2_ is essentially pure M phase when 1,2-dichloroethane is used as an additive. The curves (b–h) are the Raman spectra of ZrO_2_ NPs prepared using diethanolamine and 1,2-dichloroethane as additives, in the stipulated ratios. As shown in Figure 2b–h, the vibrational mode intensity of the T phase of ZrO_2_ gradually decreases, whereas the vibration mode of M phase of ZrO_2_ gradually increases, with decreasing diethanolamine and increasing 1,2-dichloroethane. This indicates that the results of the Raman and XRD analyses are consistent.

According to a previous report [37], the proportion of specific crystal forms of molecules can be estimated by using the characteristic bands of different crystal phases in the Raman spectra of molecules. As is illustrated in Figure 2 (curves a and i), the T and M phases of ZrO_2_ can be identified by the most representative Raman displacement located at the following positions: T (145, 266, and 646 cm^−1^) and M (177, 188, and 612 cm^−1^). The T phase content in ZrO_2_ was calculated by the relative intensities of the T bands located at 145, 266, and 646 cm^−1^, and the M bands located at 177, 188 and 612 cm^−1^. The ratios of the T phase of ZrO_2_ were calculated using the empirical expressions of Clarke and Adar [38]. The formula is as follows (3):

(3) $$\nu_{t}=\frac{0.97[I_{t}(145)+I_{t}(266)+I_{t}(646)]}{0.97[I_{t}(145+I_{t}(266)+It\left(646\right)]+[I_{m}(177)+I_{m}(188)+I_{m}(612)]}$$

where m and t represent the M and T phases, ν_t_ is the ratio of the T phase of ZrO_2_, I_m_ is the intensity of the M phase of ZrO_2_, and I_t_ is the intensity of the T phase of ZrO_2_, respectively. The ratio of the T phase in ZrO_2_ NPs is: 99.6%, 82.0%, 70.1%, 63.4%, 41.5%, 29.7%, 19.0%, 11.5%, and 2.6% corresponding to a–i in Figure 2, respectively. These calculation results are in good agreement with those calculated using the characteristic diffraction peaks of XRD.

### 3.3. UV-Vis DRS Spectra of ZrO_2_ NPs

**Figure 3:**
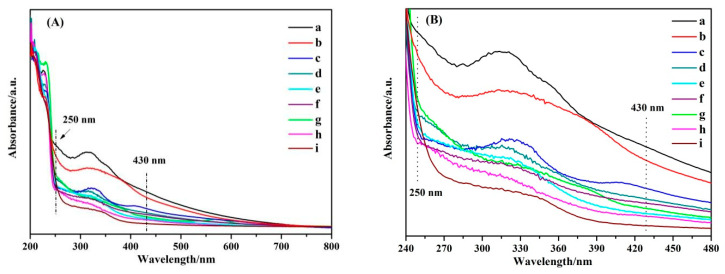
(**A**) UV-Vis DRS spectra of ZrO_2_ NPs with different T phase proportions; (**B**) UV-Vis DRS spectra of ZrO_2_ NPs with different T phase proportions in the range 240–480 nm.

The optical properties of ZrO_2_ NPs were studied by the UV-Vis DRS technique. Figure 3 shows the absorbance spectra of ZrO_2_ at different crystal ratios. The UV-Vis absorption spectrum of the semiconductor can determine its band gap energy and surface defect state [39]. The strong absorption band of nine samples at less than 250 nm is due to the band-band energy transition of ZrO_2_ NPs. The trailing absorption bands at 250–430 nm are assigned to the surface defect state of ZrO_2_. The optical band gap and incident photon energy correspond to the transformed Kubelka–Munk function [40]. The calculated value of the band gap energy (Eg(eV))and the photo-absorption thresholds (λ_g_(nm)) can be achieved from the Figure S2 and Table S2 of the Supplementary Information. Thus, the Eg with λ_g_ of ZrO_2_ which corresponds to the curves of a–i are 2.96 (419), 3.16 (392), 3.41 (364), 3.66 (339), 3.81 (325), 3.95 (314), 4.22 (294), 4.41 (281), and 4.68 eV (265 nm), respectively [41]. It is noteworthy that with the increase of the T phase ratio in ZrO_2_, the λ_g_ of band-band Transition for ZrO_2_ are red shifted, and the intensity of the tail absorption band at 250–430 nm is gradually enhanced. The greater the red shift of the optical absorption threshold, the higher the threshold, the smaller its band gap energy. The greater the strength of the trailing absorption bands indicates the stronger the surface defect strength. When the ratio of the T crystal phase in ZrO_2_ is the greatest, the absorbance bands assigned to the ZrO_2_ have the largest red shift and the strongest strength of the trailing absorption band, thus indicating that it has the best abundant surface properties. The smaller the band gap energy and the better abundant surface defect state of the semiconductor, the more favorable the electron transfer.

### 3.4. Crystal Form-Dependence of SERS

**Figure 4:**
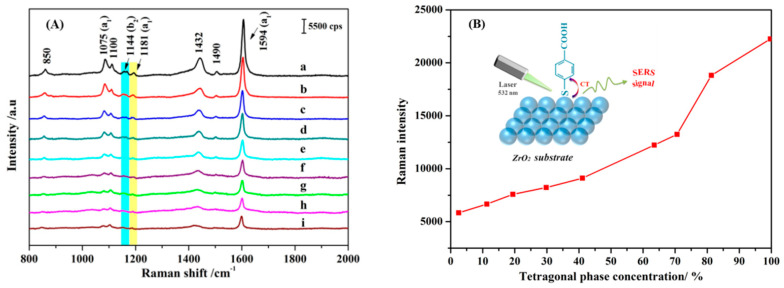
(**A**) SERS spectra of 4–MBA adsorbed on ZrO_2_ NPs with different T phase proportions. (**B**) The relationship between SERS intensities of 4–MBA at the 1594 cm^−1^ mode and the T phase concentration in ZrO_2_ NPs.

Generally, ZrO_2_ have M, T, and C crystal forms, and the different crystal forms have different grain boundary structures. However, the structure of the grain boundary will affect the electrical and optical properties of ZrO_2_. To examine the effect of the crystal form of ZrO_2_ on the SERS, Figure 4A shows the SERS spectra of 4–MBA adsorbed on ZrO_2_ NPs with different T phase proportions. Notably, as the T phase ratio increases and the M phase ratio decreases, the SERS signal of 4–MBA adsorbed on the ZrO_2_ NPs gradually increases. When the ratio of the T phase in ZrO_2_ NPs is close to that of the pure T phase, the SERS signal is at maximum. When the ratio of the M phase in ZrO_2_ NPs is close to that of the pure M phase, the SERS intensity decreases significantly. The strongest Raman intensity was obtained from the pure T phase, which is approximately four times that of the pure M phase (Figure 4B). Thus, the interdependency between the form of the crystalline phase in ZrO_2_ NPs and the SERS enhancement effect can be confirmed. The T phase of ZrO_2_ favors CT in the SERS enhancement more than the M phase. This is because of the effect of the grain boundary structure of the different crystalline phases on the electrical and optical properties of ZrO_2_. In addition, the T crystalline phase of ZrO_2_ has more abundant surface states, which is more conducive to the occurrence of CT.

### 3.5. Evaluation of EF

**Figure 5:**
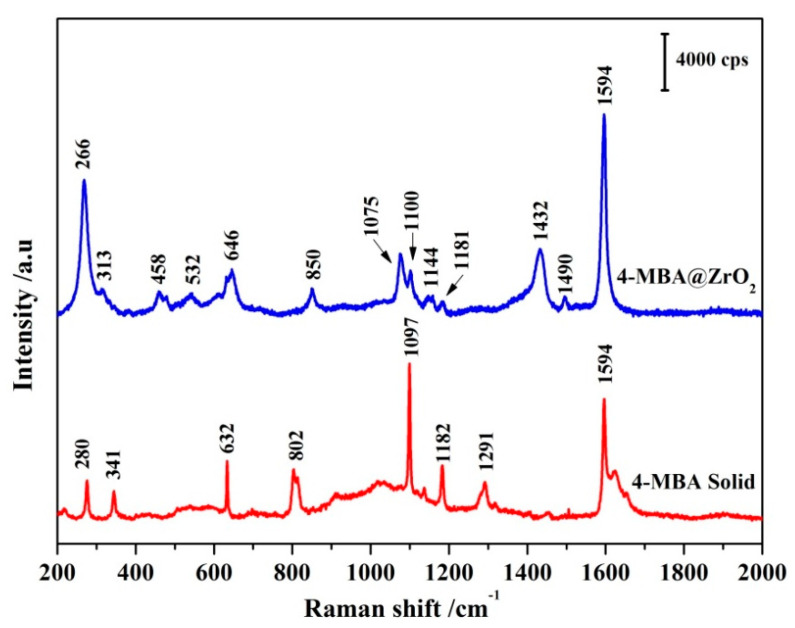
SERS spectra of 4–MBA adsorbed on ZrO_2_ NPs and Raman spectra of the 4–MBA bulk sample.

To evaluate the SERS activity of ZrO_2_ NPs, EF was employed to reflect the enhancement ability. To accurately calculate the values of EF, the Raman spectra of 4–MBA adsorbed on ZrO_2_ NPs, and of the solid 4–MBA powders were obtained as shown in Figure 5. The calculation method adopts Formula (4) [42]:

(4) $$\mathrm{EF}=\frac{I_{\mathrm{Surf}}}{I_{\mathrm{Bulk}}}\times \frac{N_{\mathrm{Bulk}}}{N_{\mathrm{Surf}}}$$

where I_surf_ and I_Bulk_ represent the SERS intensity of 4–MBA adsorbed on the ZrO_2_ NPs and 4–MBA solid powders at 1594 cm^−1^ (assigned to the characteristic vibration of the ν(C–C) aromatic ring), respectively. N_Surf_ and N_Bulk_ represent the number of molecules adsorbed on ZrO_2_ NPs and 4–MBA solid powders, respectively.

The value of I_surf_/I_Bulk_ is equal to the Raman intensity ratio of the 4–MBA@ZrO_2_ to the bulk 4–MBA in Figure 5, and the calculated result is 1.8. N_Bulk_ (the number of bulk 4–MBA detected in the 1.3 μm laser spot) was estimated to be 7.61 × 10^10^ based on 532 nm laser excitation, the focusing penetration depth of the laser (~10 μm), and the density of the 4–MBA (1.5 g·cm^−3^) [43]. If the ZrO_2_ NPs are assumed to be uniformly distributed in a single layer, and the boundary density of 4–MBA adsorbed on the NPs surface is 0.5 nmol/cm^2^, the calculated N_Surf_ is 6.146 × 10^6^ [43]. The results of the above calculation are substituted into Formula (4), and the value of EF is 2.21 × 10^4^.

### 3.6. Degree of Charge Transfer

**Figure 6:**
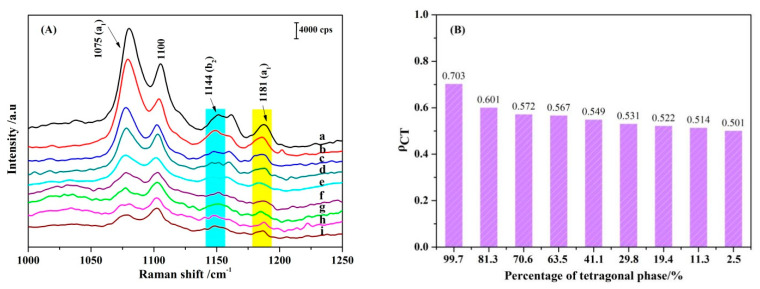
(**A**) SERS spectra of 4–MBA adsorbed on ZrO_2_ NPs with different T phase proportions. (**B**) Degree of charge transfer (ρ_CT_) values of the different T phase proportions of ZrO_2_ NPs based on 532 nm laser excitation.

CT is also the major contribution to SERS in addition to surface plasmon resonance (SPR) and molecular transition [44]. In accordance with previous reports, we employed the degree of CT (ρ_CT_(k)) to measure the CT contribution to SERS. The concept of ρ_CT_(k) can be defined by the following Formula (5) [45]:

(5) $$\rho \mathrm{CT}\left(k\right)=\frac{I^{k}\left(\mathrm{CT}\right)-I^{k}\left(\mathrm{SPR}\right)}{I^{k}\left(\mathrm{CT}\right)+I^{0}\left(\mathrm{SPR}\right)}$$

where k refers to a specific band in the Raman spectrum. In this equation, I^k^(CT) and I^k^(SPR) are the intensities of the k band in the presence of CT and EM contribution, respectively, whereas I^0^(SPR) is the intensity of the fully symmetric vibrational mode, selected in the spectral region with only SPR contribution. k can be a fully symmetric or non-fully symmetric line. For a fully symmetric line, I^k^(SPR) = I^0^(SPR), but in the latter, I^k^(SPR) is usually quite small [46,47], and we can think of it as being negligible, that is, I^k^ (SPR) = 0. It can be seen from Formula (5) that when ρ_CT_ = 0, there is no CT contribution, and when ρ_CT_ = 1, the intensity of SERS mainly comes from the CT effect. When ρ_CT_ = 1/2, the contribution of CT and EM is equal. As shown from Figure 6A, because the peak at 1144 cm^−1^ is an independent b_2_ vibrational mode, it is selected as the research object of CT contribution, that is, I^k^ (CT). The frequency band at 1181 cm^−1^ is an independent a_1_ vibrational mode, which is selected as I^0^ (SPR). After selecting the contribution peaks of the corresponding objects, the relationship of ρ_CT_ and the ZrO_2_ NPs with different T phases were shown in Figure 6B. The ρ_CT_ value indicates that when the ratio of diethanolamine to 1,2-dichloroethane is 1:0, and the purity of T zirconia is 99.7%, the CT degree is the strongest. With a decrease in purity, the CT degree gradually decreases, and all of them are greater than 0.5, indicating that CT is the main contributor to SERS intensity.

### 3.7. SERS Mechanisms of ZrO_2_ NPs

The enhancement mechanism of SERS has always been a core contentious issue in the research of SERS substrate development. The study of the SERS enhancement mechanism provides theoretical guidance for the development of high-quality SERS substrates. There are two widely accepted SERS mechanisms to expound the SERS influence. One is EM and the other is CM. The EM effect is due to the local electric field generated by the collective oscillation of the plasma on the metal surface [48,49]. The CM effect is because the CT process between the substrate and the molecule can induce CM. The CT processes typically occur between metal or semiconductor substrates, and the probe molecules. As the SPR is some distance away from the visible region, the SERS effect of most semiconductor materials is due to the CT between semiconductor materials and molecules. As shown in 4–MBA@ZrO_2_ of Figure 5, the Raman spectra of 4–MBA on ZrO_2_ are similar to those of 4–MBA on other semiconductor materials reported in the literature. Two stronger Raman bands at 1075 and 1594 cm^−1^ are attributed to the ν_12_ (a_1_) and ν_8a_ (a_1_) modes of the respiratory vibration in the aromatic ring, respectively. The other two weaker Raman bands at 1144 and 1181 cm^−1^ are assigned to the ν_15_ (b_2_) and ν_9_ (a_1_) [50,51] modes of the bending deformation vibration, respectively.

**Figure 7:**
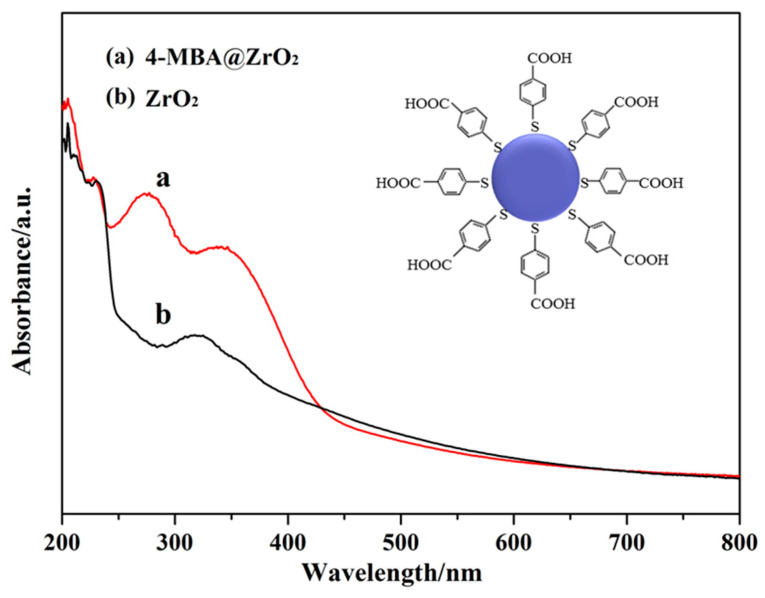
UV-Vis DRS spectra of (**a**) 4–MBA adsorbed on the ZrO_2_ NPs and (**b**) ZrO_2_ NPs (the ratio of T phase is 99.7%). The inset is the schematic diagram of 4–MBA adsorbed on ZrO_2_ NPs.

In contrast, it can be seen from the comparison of the UV absorption spectra of ZrO_2_ and 4–MBA@ZrO_2_ (Figure 7), that after 4–MBA adsorption, the intensity of the band energy transition absorption band of ZrO_2_, at less than 250 nm, has increased, and the red shift is significant. At 250–430 nm, the increase in the tail band intensity was greater and the degree of the red shift was larger than that at 250 nm. These changes indicate a CT process between the 4–MBA and the ZrO_2_ NPs [52]. The obvious changes in the tail band intensity and the degree of the red shift of the bands at 250–430 nm indicated that the surface state energy level, caused by surface defects, plays an important role in the CT process. The degree of surface defects directly affects the CT process and eventually leads to the SERS effect. Similar results were found in the Cu_2_O–4–MBA system [53].

**Figure 8:**
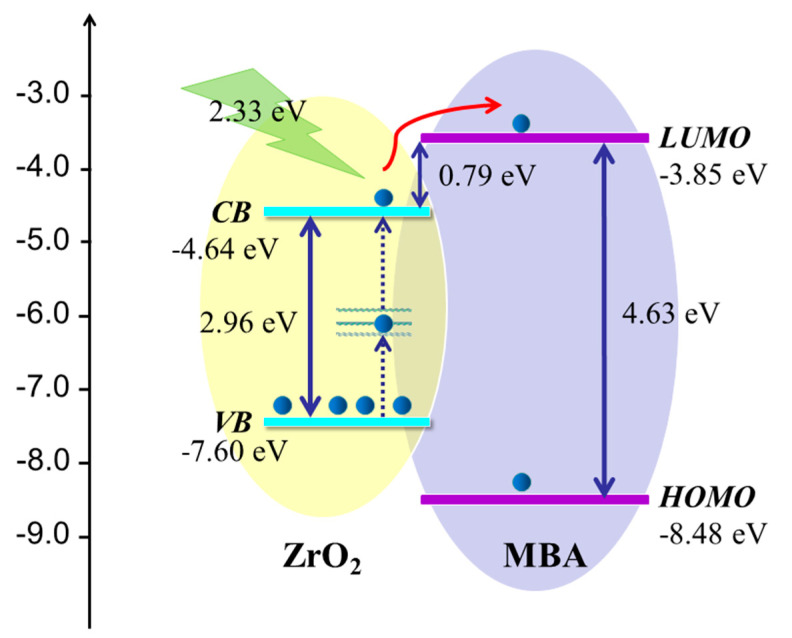
Diagram of the CT mechanism of 4–MBA–ZrO_2_ system.

The CT process in the SERS system of a semiconductor-molecule depends on the vibration coupling between the semiconductor and molecular energy levels. The direction of CT relies on the energy levels of the valence (VB) and conduction bands (CB) of the semiconductor, relative to the highest occupied molecular orbitals (HOMO), and the lowest unoccupied molecular orbitals (LUMO) of the adsorbed molecules. The HOMO and LUMO of the adsorption molecule 4–MBA were −8.48 and −3.85 eV, based on previous literature [54,55]. The VB of ZrO_2_ is located at −7.6 eV [56]. As the band gap energy of ZrO_2_ is 2.96 eV, the calculated CB of ZrO_2_ is located at −4.64 eV, (Figure 8). As ZrO_2_ with a large amount of oxygen vacancies enriching the surface states, there are transition energy levels (surface energy levels) between the VB and CB of ZrO_2_. A possible CT transfer pattern is shown in Figure 8; incident light of 532 nm (2.33 eV) excites electrons from the VB of the ZrO_2_ through the surface state (Ess) energy level to the CB of ZrO_2_, then transits to the LUMO of the 4–MBA, and finally radiates out the Raman photons. The surface defects can be explained by using the UV-Vis DRS spectra. It can be seen from the curve i–a in the Figure 3 that with the increase of the T phase ratio in ZrO_2_, the intensity of the tail absorption band at 250–430 nm is gradually enhanced, indicating that the degree of surface defect state gradually increases. The rich surface defect state is more beneficial to the SERS effect during CT transfer. With the increase of the T crystal form ratio, more surface defects are produced, which lead to the stronger SERS effect. The experimental results are consistent with the above enhancement mechanism, which indicates that the SERS effect of the ZrO_2_ substrate can be improved by adjusting the ratio of the T crystalline phase in the ZrO_2_.

## 4. Conclusions

ZrO_2_ NPs with different ratios of the crystalline phase were synthesized by hydrothermal synthesis and adjusting the ratio of two additives. The additive, diethanolamine, encourages the formation of T–ZrO_2_ crystals, whereas 1,2-dichloroethane encourages the formation of M–ZrO_2_ crystals. The ratio of the T and M phases of ZrO_2_ NPs can be easily controlled. When the probe molecules of 4–MBA are adsorbed onto the ZrO_2_, the SERS intensity of 4–MBA is gradually enhanced with the increasing proportion of the T phase of ZrO_2_ NPs. The strongest SERS intensity of 4–MBA can be obtained by using the pure T phase. The value of EF reached 2.21 × 10^4^. The UV-Vis DRS spectra have shown the interrelation between the band gap energy, surface defect state and the proportion of T phase in ZrO_2_ NPs. As the proportion of T phase in ZrO_2_ NPs increases, the band gap energy decreases but the degree of surface defects increases gradually. The smaller band gap energy and the richer surface defect states are conducive to CT transfer between ZrO_2_ NPs and 4–MBA, consequently showing much stronger SERS signal. This is the main reason that the SERS effect of the T–ZrO_2_ substrate is stronger than that of the M–ZrO_2_ substrate. The schematic diagram of CT shows the direction of CT and further indicates that the crystal form of ZrO_2_ NPs is an important factor affecting the CT. The SERS effect of semiconductor substrates was examined from the perspective of the crystal form of semiconductor nanomaterials in an unprecedented attempt. This study illustrates a new way to develop better quality semiconductor substrates and highlights a new method to improve the charge transport rate for the construction of high sensitivity molecular sensors.

## Data Availability

Do not have the supporting reported results.

## Supplementary Materials

The following are available online at https://www.mdpi.com/article/10.3390/nano11092162/s1, Figure S1: SEM images of ZrO_2_ NPs prepared with different ratios of diethanolamine and 1,2-dichloroethane as mixed additives. a–i correspond to mixing ratio of diethanolamine and 1,2-dichloroethane are 1:0, 4:1, 3:1, 2:1, 1:1, 1:2, 1:3, 1:4, and 0:1, respectively, Figure S2: UV-Vis DRS spectra of ZrO_2_ NPs prepared with different ratios of diethanolamine and 1,2-dichloroethane as mixed additives. a–i correspond to mixing ratio of diethanolamine and 1,2-dichloroethane are 1:0, 4:1, 3:1, 2:1, 1:1, 1:2, 1:3, 1:4, and 0:1, respectively.
