# Supplementary material for: Surface-Enhanced Raman Scattering Activity of ZrO2 Nanoparticles: Effect of Tetragonal and Monoclinic Phases
Source: Nanomaterials (Basel). 2021 Aug 24;11(9):2162. doi: 10.3390/nano11092162 (PMC8466494; doi:10.3390/nano11092162)
Supplement: Supplementary file 1 [file nanomaterials-11-02162-s001.zip › nanomaterials-1343178-supplementary.pdf]

nanomaterials

Supplementary Materials

Surface-Enhanced Raman Scattering Activity of ZrO_2 Nanoparticles: Effect of Tetragonal and Monoclinic Phases

Mingyue Yi [1], Yu Zhang [1], Jiawen Xu [1], Dingyuan Deng [1], Zhu Mao [2], Xiangchun Meng [1], Xiumin Shi [1,*] and Bing Zhao [3,*]

[1] College of Chemical Engineering, Changchun University of Technology, Changchun 130012, China; yimingyue95@163.com (M.Y.); zhangyjy0@163.com (Y.Z.); xujiawen20210719@163.com (J.X.); dengdingyuan0824@163.com (D.D.); mengxiangchun@ccut.edu.cn (X.M.)
[2] School of Chemistry and Life Science, Changchun University of Technology, Changchun 130012, China; maozhu@ccut.edu.cn
[3] State Kay Laboratory of Supramolecular Structure and Material, Jilin University, Changchun 130012, China
* Correspondence: shixiumin@ccut.edu.cn (X.S.); zhaob@jlu.edu.cn (B.Z.); Tel.: +86-431-85716463 (X.S.)

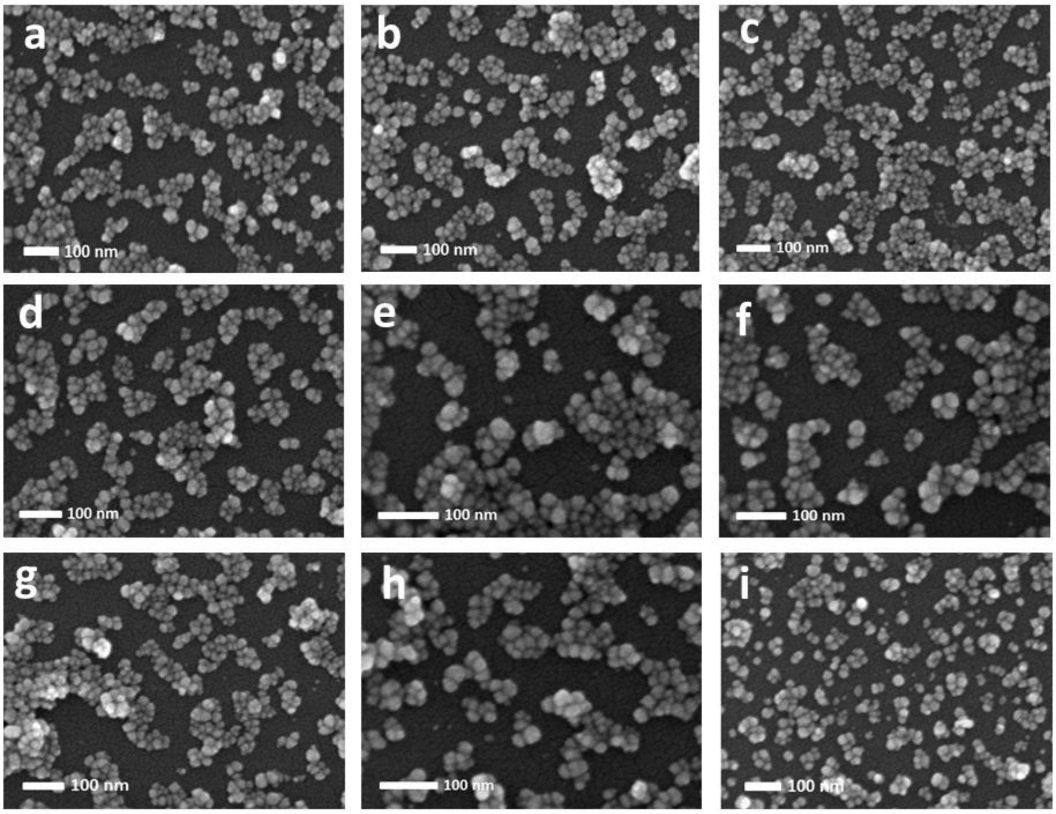

Figure S1. SEM images of ZrO_2 NPs prepared with different ratios of diethanolamine and 1,2–dichloroethane as mixed additives. a, b, c, d, e, f, g, h, and i correspond to mixing ratio of diethanolamine and 1,2–dichloroethane are 1:0, 4:1, 3:1, 2:1, 1:1, 1:2, 1:3, 1:4, and 0:1, respectively.

Table S1. The average particle sizes of the ZrO_2 NPs with different T phase proportions.

Curve in the figure S1	a	b	c	d	e	f	g	h	i
ZrO_2 of different dispersant proportions	1:0	4:1	3:1	2:1	1:1	1:2	1:3	1:4	0:1
ZrO_2 of different T phase proportions/ %	99.7	81.3	70.6	63.5	41.1	29.8	19.4	11.3	2.5
Size/nm	16.5	15.6	15.5	15.9	15.7	15.6	16.3	16.4	16.7

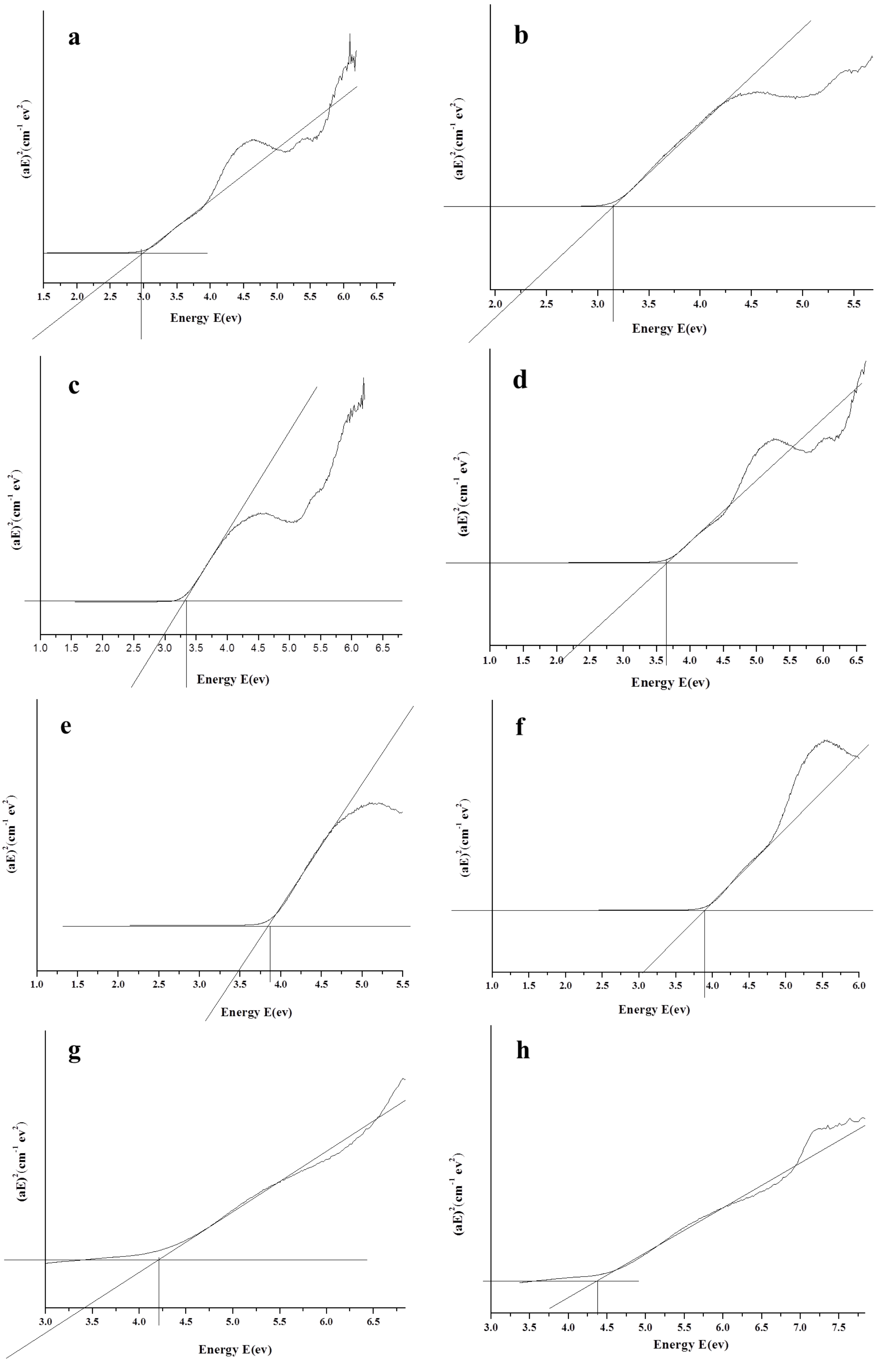
a
b
c
d
e
f
g
h
(aE)^2(cm^-1 ev^2)
Energy E(ev)

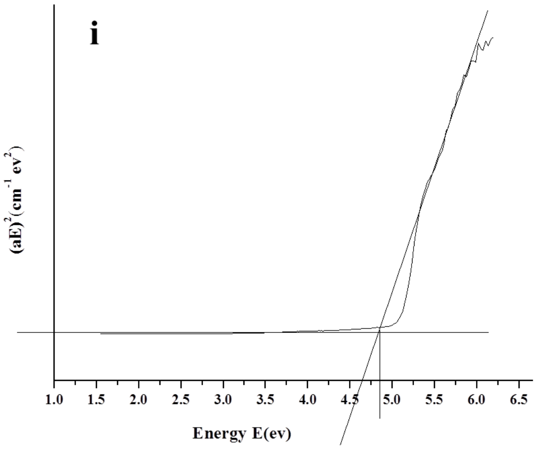

Figure S2. UV-Vis DRS spectra of ZrO_2 NPs prepared with different ratios of diethanolamine and 1,2–dichloroethane as mixed additives. a, b, c, d, e, f, g, h, and i correspond to mixing ratio of diethanolamine and 1,2–dichloroethane are 1:0, 4:1, 3:1, 2:1, 1:1, 1:2, 1:3, 1:4, and 0:1, respectively.

The calculated value of the band gap energy (E_g(eV)) and the photoabsorption thresholds (λg (nm)) can be achieved from the Figure S2 and Table S2. The λg can be obtained according to the formula: $E_g = \frac{1240}{\lambda_g}$

Table S2. The band gap energy (Eg) and the photoabsorption thresholds (λ_g) of band-band transition for ZrO_2 NPs with different T phase proportions.

**Curve in the figure 3 (B)**	**a**	**b**	**c**	**d**	**e**	**f**	**g**	**h**	**i**
ZrO_2 of different T phase proportions/ %	99.7	81.3	70.6	63.5	41.1	29.8	19.4	11.3	2.5
the band gap energy of ZrO_2/ eV	2.96	3.16	3.41	3.66	3.81	3.95	4.22	4.41	4.68
λ_g/ nm	419	392	364	339	325	314	294	281	265
